# CRISPR-based knockout screening identifies the loss of MIEF2 to enhance oxaliplatin resistance in colorectal cancer through inhibiting the mitochondrial apoptosis pathway

**DOI:** 10.3389/fonc.2022.881487

**Published:** 2022-08-29

**Authors:** Chaozheng Xie, Kang Li, Ya Li, Xudong Peng, Biyun Teng, Kuan He, Aishun Jin, Wang Wang, Zhengqiang Wei

**Affiliations:** ^1^ Department of Gastrointestinal Surgery, The First Affiliated Hospital of Chongqing Medical University, Chongqing, China; ^2^ Department of Endocrine and Breast Surgery, The First Affiliated Hospital of Chongqing Medical University, Chongqing, China; ^3^ Department of Vascular Surgery, The First Affiliated Hospital of Chongqing Medical University, Chongqing, China; ^4^ Chongqing Key Laboratory of Basic and Translational Research of Tumor Immunology, Chongqing Medical University, Chongqing, China

**Keywords:** CRISPR screens, oxaliplatin, colorectal cancer, MIEF2, mitochondrial apoptosis

## Abstract

The first-line anticancer agent oxaliplatin (OXL) is the preferred drug for treating colorectal cancer (CRC); however, the development of drug resistance is common in patients treated with OXL, which considerably reduces the efficacy of OXL-based regimens. By performing genome-wide CRISPR/Cas9 library knockdown screening, we found that mitochondrial elongation factor 2 (MIEF2) was among the top candidate genes. The OXL-resistant cell lines and organoids developed in the present study showed stable but low expression of MIEF2. Reduced MIEF2 expression may enhance CRC resistance to OXL by reducing mitochondrial stability and inhibiting apoptosis by decreasing cytochrome C release. In conclusion, among the different biomarkers of OXL resistance in CRC, MIEF2 may serve as a specific biomarker of OXL responsiveness and a potential target for the development of therapies to improve chemotherapeutic effectiveness.

## Introduction

Colorectal cancer (CRC) is one of the most common malignant tumors, and its morbidity and mortality are among the highest of all malignant tumors, with an increasing in young individuals (1). Although the survival rate of patients with early-stage CRC is better, the survival rate of those with middle- and late-stage CRC remains low as the disease is difficult to treat in the advanced stages (2). Oxaliplatin (OXL), a third-generation platinum compound, is a first-line chemotherapeutic agent used for the treatment of CRC (3) and is also the only active platinum drug approved for treating CRC. Adding OXL to CRC chemotherapy regimens has been reported to significantly improve patient prognosis (4). However, only a small proportion of patients have been reported to exhibit objective remission to standard chemotherapy, and an important reason for treatment failure is tumor intrinsic or acquired resistance (5). Recent studies have suggested that multiple molecular mechanisms may be simultaneously involved in the development of OXL resistance (6–9) such as intracellular transport and detoxification, altered repair mechanisms after DNA damage, cell death mechanisms, and epigenetics. Among all these molecular mechanisms, apoptosis plays a major role in response to cancer therapy, and altered susceptibility to apoptosis critically determines the response to chemotherapy treatment (10). Therefore, a fundamental goal for more effective CRC treatment is to gain a deeper understanding of the mechanism of action and resistance to OXL.

A few researchers have developed the genome-scale CRISPR screening technology based on the CRISPR/Cas9 gene-editing technology (11, 12), which is mostly used for genetic screening of cancer drugs and their effects (13–16). Therefore, we performed genome-wide CRISPR/Cas9 knockdown screening to identify key genes that contributed to overcoming OXL resistance in CRC cells. We performed a follow-up validation study based on the screening results and identified mitochondrial elongation factor 2/mitochondrial dynamics proteins of 49 kDa (MIEF2/MID49) as a candidate target. MIEF2 encodes an outer mitochondrial membrane protein that directly recruits the fission mediator dynamin 1 like (DNM1L) to the mitochondrial surface, and its function is to regulate mitochondrial morphology (17–19). The depletion of MIEF2 eliminates DNM1L oligomerization in the mitochondria, leading to mitochondrial elongation rate or collapse (20). Notably, the mitochondria are also essential in regulating apoptotic cell death (21, 22), wherein mitochondrial dynamics are closely related to apoptosis. It is generally believed that mitochondrial fission accelerates apoptosis, whereas mitochondrial elongation protects cells from death (23). The mitochondria are a potential target for improving OXL efficacy and reducing its toxicity (24). MIEF2 knockdown (KO) has been reported to protect cells from intrinsic apoptotic progression *via* mitochondrial cristae remodeling (25), and we believe that MIEF2 plays an important role in OXL-mediated mitochondrial apoptosis and that its depletion increases resistance to OXL *via* the inhibition of apoptosis.

In the present study, we validated 3726 genes involved in OXL-resistant CRC-SW480 cells by genome-scale CRISPR-Cas9 knockout screening, and some growth-essential genes and protective molecules. We identified the deletion of MIEF2 as a key driver of OXL resistance in CRC. OXL treatment induces apoptosis and damages the mitochondria through the mitochondrial apoptosis pathway. During OXL treatment, we detected the deletion of MIEF2, which inhibited apoptosis by affecting mitochondrial stability and reducing cytochrome C release, eventually worsening OXL resistance.

## Materials and methods

### Ethics statement

The studies involving human participants were reviewed and approved by the Ethical Committee of the First Affiliated Hospital of Chongqing Medical University (2020-557). The patients/participants provided their written informed consent to participate in this study.

### Cell line

The CRC cell line SW480 was sourced from the American Type Culture Collection and was regularly maintained in our laboratory. SW480 and its derived oxaliplatin resistant (SW480-OXR) cells as well as knockout clones were cultured in the RPMI 1640 Medium (Gibco, cat. no. C11875500BT) supplemented with 10% fetal bovine serum (FBS) (BI, cat. no. 04-001-1ACS) and 1% penicillin and streptomycin (Gibco, cat. no. 15140122). SW480-OXR were established by treating the cells with an initial high dose (10 μM) of oxaliplatin (Selleck, cat. no. S1224) during the first week, followed by exposure to 5 μM oxaliplatin for 4 weeks after cell recovery. The resistant cells were maintained in a medium containing 1 μM oxaliplatin and restored to a complete medium without oxaliplatin 2 days before conducting the experiments.

### Organoid acquisition

The CRC organoids were obtained as generous gifts from the Immunology Research Center of Chongqing Medical University, Chongqing, China.

### Drug selection pressure and cell viability assay

We performed a dose-response curve analysis of the drug to determine the optimized selection pressure, that is, the minimal lethal dose (MLD) that could kill all control cells before their screening. For OXL, a selection pressure of 12 μM concentration was selected. For the cell viability assay, 1000-3000 cells were inoculated into a 96-well plate, and, once attached, the cells were treated with OXL for 48 h. The medium was aspirated and replaced with a fresh medium without drug exposure, and the cell viability was measured using the Cell Counting Kit-8 (CCK8) (MCE, cat. no. HY-K0301). The organoid viability was assessed using the CellTiter-Glo^®^3D Cell Viability Assay (Promega, cat. no. G9683). The drug response curve for OXL was plotted and the half-inhibitory concentration (IC50) was calculated using the GraphPad Prism version 8.3.0 (GraphPad Software, San Diego, CA, USA).

### Plasmid and library screening

The principle of CRISPR-Cas9-mediated knockdown is that the endonuclease Cas9 can be directed to specific genomic targets *via* sgRNA to produce double-stranded breaks and loss-of-function mutagenesis. Human Brunello CRISPR knockout pooled library was obtained as a gift from David Root and John Doench (Addgene #73179), which contained 76,441 gRNAs targeting 19,114 genes in the human genome with higher activity and targeting (26). For this screen, we chose the single plasmid system of Brunello library, where each plasmid backbone contains SpCas9 genetic information and a unique sgRNA. First, we packaged the plasmid into lentiviral particles using polyethylenimine, branched (PEI) (Sigma-Aldrich, cat. no. 408727) reagent, and 293 T cells. The constructed Brunello lentiviral library was transduced into the CRC cell line SW480 by transducing the lentiviral library at a low multiplicity of infection (MOI = 0.3) (as shown in **Supplementary Figure S1B**: **Figure S1B**). After 24 h of infection, we used a medium containing 1 μg/mL puromycin to select the transduced cells for 7 days to obtain mutant cells pool. 4×10 (7) mutant cells were collected as the Baseline group. Then the same number of mutant cells were taken and treated with vector (PBS group) and 10 μM oxaliplatin (OXL group) for 10 d, respectively. Subsequently, we extracted gDNA and amplified the sgRNA region by polymerase chain reaction (PCR) with the lowest cycle number (24), followed by deep sequencing (Hiseq-PE150, from Novogene Genetics, China) (**Figure S1D**). MAGeCK v0.5.7 algorithm was used to analyze the hit sgRNA counts (27, 28).

### Database analysis of MIEF2 expression in CRC

The relationship between MIEF2 expression and clinicopathological features in CRC was analyzed using UALCAN (http://ualcan.path.uab.edu), with data from The Cancer Genome Atlas (TCGA).

### Generation of MIEF2-knockdown cells/organoids and MIEF2-overexpression cells

Short hairpin RNAs (sh) shMIEF2-1 and shMIFE2-2 targeting MIEF2 were designed, and their sequences are shown in **Supplementary File 1**: **Table S1**. They were synthesized by Tsingke (Beijing, China) and cloned into pLKO.1 with modified enzyme sites BamHI and EcoRI.The sgRNA sequences targeting MIEF2 were retrieved from the Brunello library (**Table S1**). The plasmid used to construct the sgRNA encoding was lentiCRISPR v2 (Addgene #52961) was provided by Feng Zhang’s laboratory. To generate MIEF2-knockdown cells and organoids, the above plasmids were transduced into CRC cell lines and organoids using Lipofectamine^®^ 3000 (Invitrogen). The MIEF2 DNA fragment was generated by PCR and cloned into pCDH-vector (**Table S2**). CRC cell lines were transfected with either empty pCDH-vector or with the MIEF2-pCDH-vector using Lipofectamine^®^ 3000.

Transduced cells were selected using puromycin-containing medium, followed by isolation and expansion of single cells, and detection of MIEF2 expression by qPCR and western blotting. Cells were transduced with no targeting plasmid or empty pCDH-vector as control.

### Quantitative reverse transcription-PCR (qRT-PCR)

The total RNA from the CRC organoid and the cells was extracted with TRIzol reagent (Invitrogen, cat. no.15596026), and the RNA pellet was dissolved into 20 μL RNase-Free water. Then, 1 μg RNA sample was reversely transcribed into cDNA by the RT Master Mix for qPCR (gDNA digester plus) (MCE, cat. no. HY-K0511). Subsequently, qRT-PCR was performed using the SYBR Green qPCR Master Mix (MCE, cat. no. HY-K0501) on the CFX96 Touch Real-Time PCR Detection System with the primers listed in **Table S1**. These results were calculated with the 2–ΔΔ Ct method with normalization to GAPDH (an endogenous control gene).

### Western blotting

Total protein from the cells was extracted on an ice bath using a cell lysis buffer for Western blotting and IP (containing protease and phosphatase inhibitor cocktail for general use) (Beyotime, Shanghai, China). We used the BCA Protein Assay Kit (Beyotime) to detect the protein concentration. The protein lysates were placed in 10% or 15% polyacrylamide SDS-PAGE gels and transferred onto the PVDF membranes (Millipore). These membranes were blocked with 5% nonfat milk at room temperature and appropriately diluted with primary antibodies at 4°C (Table S2), followed by overnight incubation. Next, the membranes were incubated with anti-mouse or anti-rabbit immunoglobulin conjugates for 1 h at room temperature after washing thrice in TBS containing 0.1% Tween 20. The signals were visualized using an electrochemiluminescence detection kit (Beyotime), with GAPDH serving as an endogenous control.

### Apoptosis assay

For apoptosis assay, equal numbers of cells were grown in 6-well plates (0.4 × 10 (6) cells/well) and treated with OXL over 48 h. The adhering cells were collected at the end of treatment and washed with 2% FBS in PBS. We performed flow cytometry to determine apoptosis based on the manual of the Annexin V-FITC Apoptosis Detection Kit (Beyotime). The percentages of AnnexinV-FITC/PI-stained cells were analyzed by the Flowjo software.

### Immunohistochemical (IHC) analysis

The deparaffinize slides were baked at 65°C for 2 h and then rehydrated in a series of ethanol solutions. The endogenous peroxidase activity was quenched by 3% hydrogen peroxide for 15 min. Antigen retrieval was performed by boiling the slides in citrate buffer for 2 min. Next, the slides were blocked in normal goat serum working solution for 30 min and incubated with primary antibodies (**Table S3**) overnight at 4°C. Finally, the procedure was performed using a rabbit streptavidin-biotin assay system (ZSGB BIO, Beijing, China) according to the manufacturer’s protocol. We analyzed the immunohistochemical results using the immunoreactivity scores (IRS) with the following specific scoring criteria. The intensity of immunostaining was scored as 0 (no), 1 (weak), 2 (moderate), or 3 (strong) and the percentage of positive cells was scored as 0 (<5%), 1 (5–25%), 2 (26–50%), 3 (51–75%), 4 (76–100%). Finally, the IHC score for each sample was calculated as the intensity of immunostaining score multiplied by the percentage of positive cells score. The IRS scores were independently assessed by two observers.

### Caspase-3&9 activity analysis

After treatment with oxaliplatin in the experimental and control groups, respectively, cells were collected and caspase-3/9 activity was detected using caspase-3/9 activity assay kits (Beyotime) according to the manufacturer’s protocol.

### Mitochondrial and cytoplasmic isolation

According to the manufacturer’s protocol, mitochondrial and cytoplasmic fractions of cultured cells were isolated using the Cell Mitochondrial Isolation Kit (Beyotime). Cell precipitation was collected by centrifugation at room temperature, and 2-5 × 107 cells were suspended with 1 mL of mitochondrial isolation reagent, vortexed, and incubated on ice for 10 min, followed by centrifugation at 600 × g for 10 min at 4°C. The supernatant was centrifuged again at 3500 × g for 10 min at 4°C. The precipitation was the isolated cellular mitochondria. For the supernatant (cytoplasmic fraction), the transfer was continued by centrifugation at 12,000 × g for 10 min at 4°C, and the supernatant after centrifugation was taken as the cytoplasmic protein after mitochondrial removal.

### Statistical analysis

All experimental data were organized using GraphPad or Excel. Differences between the two groups were analyzed using t-tests, and one-way analysis of variance (ANOVA) was applied to compare multiple groups. Bioinformatics analysis was performed using the R version 4.0.2. P < 0.05 was considered to indicate statistical significance (*p < 0.05, **p < 0.01, ***p < 0.001, ****p < 0.0001).

**Figure 1:**
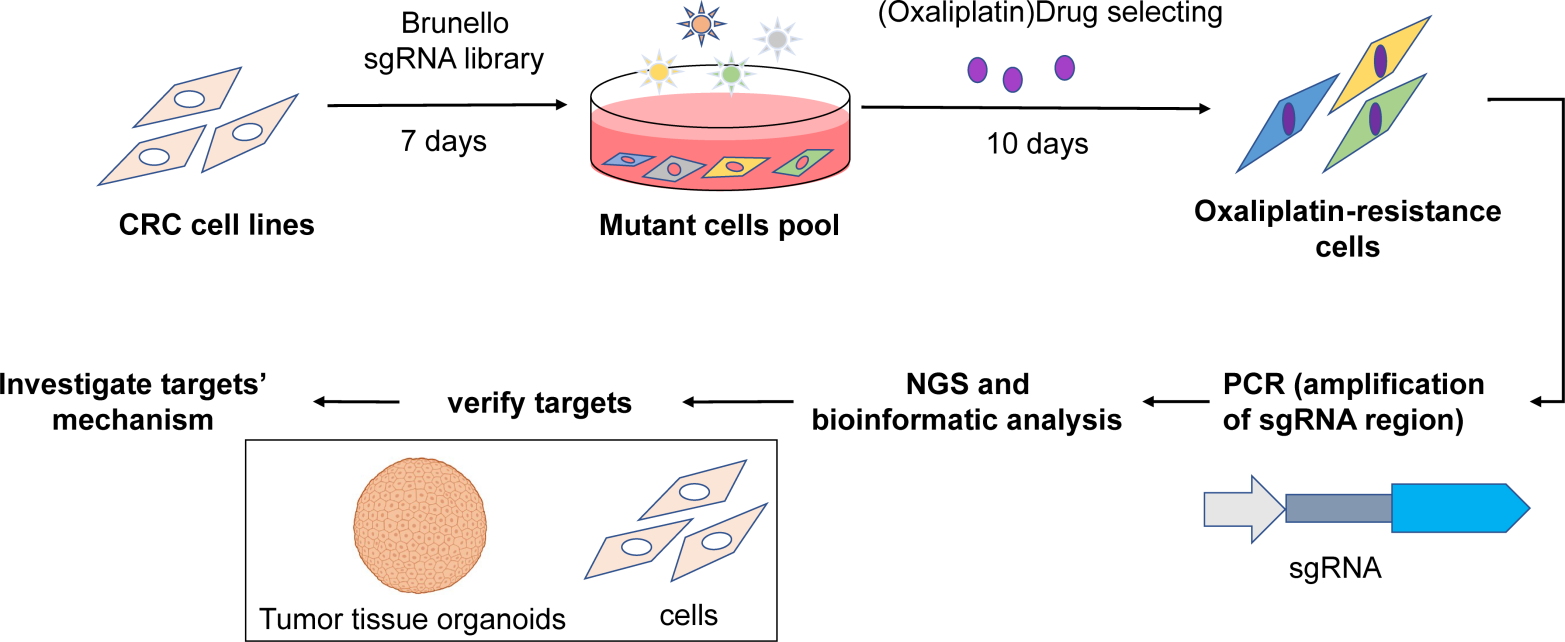
Overall workflow diagram.

The overall schematic of the screening strategy and downstream validation is illustrated in **Figure 1**.

## Results

### CRISPR library screening results of OXL in SW480 cells

To identify the key genes that regulate the response to OXL resistance in CRC, we first treated four CRC cell lines, Caco2, HT29, HCT116, SW480, with oxaliplatin, and the CCK-8 assay revealed that HCT116 and SW480 were more responsive to OXL compared to Caco2 and HT29 (**Supplementary File 1**: **Figure S1A**). Then, OXL-sensitive SW480 cells were selected for genome-wide CRISPR/Cas9 knockout library screening, and CRISPR knockout mutant cells pool was successfully constructed in SW480 with 30% lentiviral infection efficiency (**Figure S1B**). We hypothesized that the knockdown of OXL-sensitive genes would make CRC cells resistant to OXL-induced cell death or proliferation inhibition, i.e., cells carrying sgRNAs targeting OXL-sensitive genes would be positively selected in the mutant cell pool after treatment with OXL, and their corresponding sgRNAs would be enriched in the library, which could be determined by high-throughput sequencing. After 10 days of exposure to 10 μM OXL, a small population of SW480 cells infected with Brunello library lentiviruses developed resistance to OXL and still had monoclonal growth activity (**Figure S1C**). The surviving cells were harvested, the genomic DNA was extracted, the sgRNA region was amplified (**Figure S1D**), and the sgRNA was detected by high-throughput sequencing. We achieved about 500× library coverage, and about 97.20% of sgRNA sequences were retained in the baseline and PBS groups.

**Figure 2:**
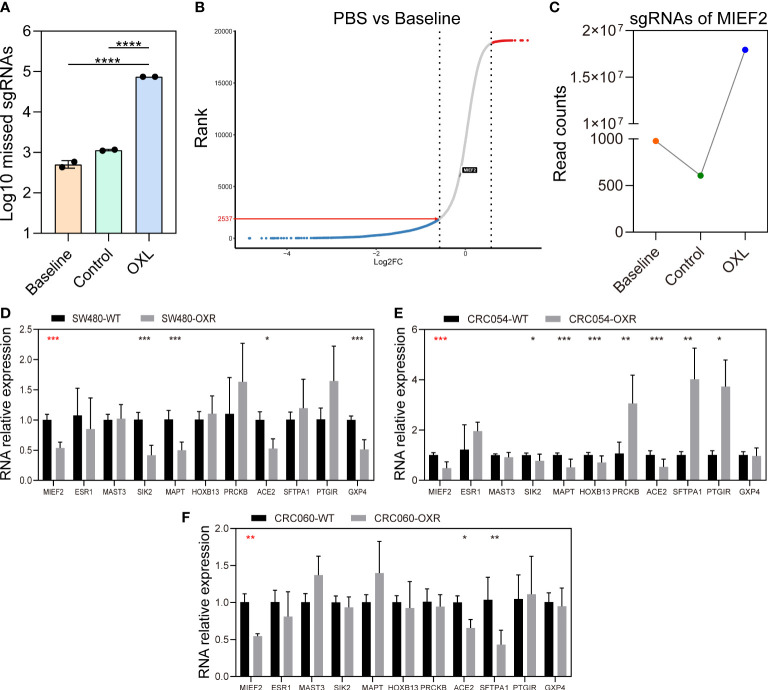
CRISPR knockout library screen identifies MIEF2 deletion as a key factor in OXL resistance. **(A)** Distribution of sgRNA in the baseline, PBS and OXL groups. **(B)** The sgRNA reads from the baseline and PBS groups were analyzed with MAGeCK and ranked genetically by robust ranking aggregation (RRA) score to check the effect of cell proliferation on library distribution. **(C)** MIEF2 was identified as the most important gene in the library screen. The sgRNA targeting MIEF2 was most enriched in the pool of OXL-treated mutant cells. **(D–F)** qPCR analysis for 11 genes in SW480, CRC054, CRC060 (OXL-sensitive), OXL-resistant cell SW480-OXR and OXL-resistant organoids CRC054-OXR and CRC060-OXR. Statistical analysis was performed using two-tailed unpaired t-test. *P < 0.05, **P < 0.01, ***P < 0.001, ****P < 0.0001 (mean ± SD, n = 3 biologically independent samples).

To explore the distribution of sgRNA libraries after OXL screening, we examined the loss of sgRNAs in the three groups (**Figure 2A**). Compared to the Baseline group, the PBS group lost sgRNAs targeting 2537 genes (**Figure 2B**), indicating that the mutant cell pool lost genes essential for cell growth after a period of culture, which is generally consistent with previous studies (29). We also examined the matches of the three sets of sgRNAs to demonstrate no significant biased selection in the screening process (**Figure S1E**).

In contrast, in the OXL group, most of the cells in this group died and carried sgRNA information that was lost after OXL screening (**Figure 2A**). Based on the CRISPR/Cas9 knockdown library screening, we identified a subset of sgRNAs targeting 3736 significantly enriched genes in OXL group (positive enrichment) (see **Supplementary File 2: Gene list of OXL**), with the highest enrichment of sgRNAs targeting MIEF2 (**Figure 2C**). In addition, the distribution of sgRNA targeting MIEF2 was not affected by the passaged growth of the mutant cells pool (**Figure 2B**).

### Construction of acquired CRC OXL drug resistance models

To validate the screening results, we constructed OXL-resistance models using the CRC cell line SW480 and organoids (CRC054 and CRC060). The morphology of OXL-resistant SW480 (SW480-OXR) cells differed from that of wild-type cells, and some cells were deformed with multinucleated giant and spindle cell formation when observed under a light microscope, whereas the organoid tended to partially dissociate after OXL treatment. However, the overall morphology remained consistent with the parental organoid (**Figure S2A**). Furthermore, the successful development of the OXL resistance model was confirmed by the increase in cell viability and IC_50_ values after OXL treatment. The average value of IC50 of OXL in SW480-OXR was approximately 70-fold higher than WT cells, approximately 97-fold higher in CRC054-OXR, and approximately 5-fold higher in CRC054-OXR, indicating that SW480-OXR, CRC054-OXR, and CRC060-OXR acquired resistance to OXL (SW480-WT vs SW480-OXR: 0.2159 μM vs 15.28 μM, CRC054-WT vs CRC054-OXR: 2.352 μM vs 229.3 μM, CRC060-WT vs CRC060-OXR: 0.5329 μM vs 3.038 μM) (**Figure S2B**).

### MIEF2 expression is negatively correlated with OXL resistance in CRC

**Figure 3:**
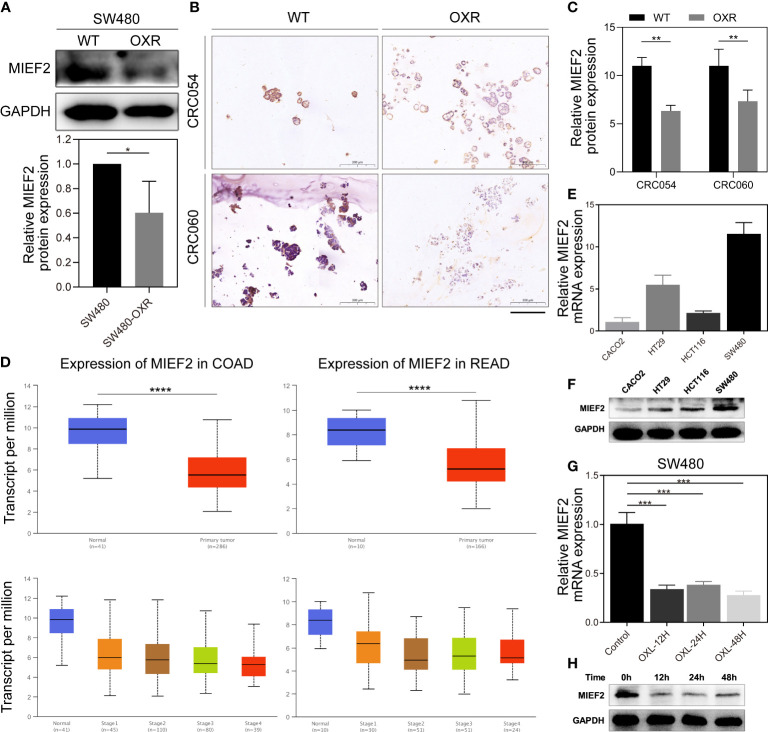
MIEF2 is involved in the CRC response to OXL. **(A)** The protein expression level of MIEF2 in SW480 and SW480-OXR was detected by western blotting analysis. Statistical analysis was performed using two-tailed unpaired t-test. *P < 0.05 (mean ± SD, n = 3 repetitions). **(B, C)** The cytoplasmic expression of MIEF2 was determined by immunohistochemical staining of paraffin-embedded sections from CRC organoids (CRC054 and CRC060) and their OXL-resistant cohort, and the immunoreactivity score (IRS) showed low expression of MIEF2 in OXR. Statistical analysis was performed using one-way ANOVA. **P < 0.01 (mean ± SD, n = 3). **(D)** MIEF2 expression in colon adenocarcinoma (COAD) and rectum adenocarcinoma (READ) was analyzed in the UALCAN database, and the results suggested that MIEF2 expression was decreased in CRC. Statistical analysis was performed using Student’s t-test. ****P < 0.0001. **(E, F)** Expression of MIEF2 in SW480 in response to OXL treatment was determined by qPCR and western blotting analysis. Induction of MIEF2 mRNA expression at each time point was expressed as means  ± SD (n  =  3). Statistical analysis was performed using two-tailed unpaired t-test. ***P < 0.001. **(G, H)** The basal expression levels of MIEF2 in CRC cell lines (Caco2, HT29, HCT116, SW480) were determined by qPCR and western blotting analysis.

To identify key genes involved in CRC response to OXL treatment, we selected the top 11 genes in the gene list and performed quantitative real-time PCR (qPCR) validation using the CRC cells and organoids. The qPCR results showed that compared with the control group, MIEF2 had a stable but low expression in SW480-OXR cells and the OXL-resistant organoids CRC054-OXR and CRC060-OXR (**Figures 2D**–**F**). We verified the differential expression of MIEF2 at the protein level, and the results showed that MIEF2 was mainly expressed in the cytoplasm and downregulated in CRC054-OXR and CRC060-OXR (**Figures 3A**–**C**).

Subsequently, we explored the distribution of MIEF2 expression in COAD and READ in TCGA. MIEF2 was significantly downregulated in tumor tissues compared to normal tissues, and the same trend existed in COAD and READ: lower expression in CRC tumors with advanced TNM stage (**Figure 3D**).

To further investigate the possible functions of MIEF2 in CRC, the expression of endogenous MIEF2 in four CRC cell lines (Caco2, HT29, HCT116, and SW480) was assessed by qPCR and western blotting analysis. The results showed that the MIEF2 mRNA levels were higher in SW480 cells and lower in Caco2 cells, with a 10-fold difference (**Figures 3E**, **F**). MIEF2 expression matched the previous CCK-8 assay results (Figure S1A), i.e., SW480 cells with higher MIEF2 expression were more sensitive to OXL treatment than Caco2 cells. In addition, to determine changes in MIEF2 expression, SW480 cells were treated with OXL and we observed that OXL treatment caused a reduction in MIEF2 mRNA levels. MIEF2 expression decreased significantly after 12 h of OXL treatment and remained low thereafter (**Figure 3G**). MIEF2 protein levels were also correspondingly low, thus confirming the previous results (**Figure 3H**). Collectively, these results suggest that MIEF2 may be involved in the CRC response to OXL.

### MIEF2 deletion increases the resistance of CRC cells to OXL

**Figure 4:**
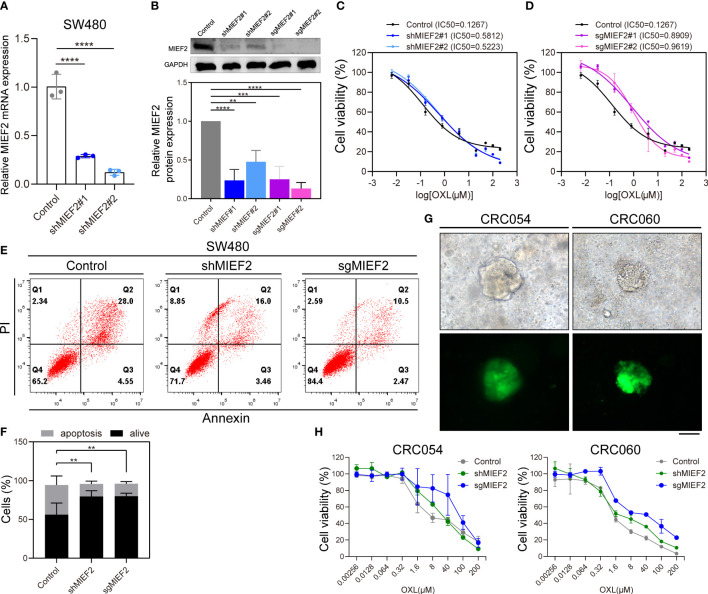
Loss of MIEF1 increases the resistance of CRC cells to OXL. **(A, B)** qPCR and western blotting to show the knockdown efficiency of shRNA and sgRNA targeting MIEF2 (shMIEF2 and sgMIEF2) in SW480. Bar plots show MIEF2 expression between different groups. Statistical analysis was performed using two-tailed unpaired t-test. **P < 0.01, ***P < 0.001, ****P < 0.001 (mean ± SD, n = 3 repetitions). **(C, D)** OXL dose-response curves of non-targeted control, shMIEF2 and sgMIEF2 (black connected dots: control; blue connected dots: shMIEF2; purple connected dots: sgMIEF2). **(E, F)** Comparison of apoptosis induction in non-targeted control and MIEF2-deficient (shMIEF2 and sgMIEF2) SW480 cells after oxaliplatin treatment by flow cytometry analysis. Knockdown of MIEF2 significantly downregulates apoptosis after OXL treatment in SW480. Stacked plots show the percentage of apoptotic (AnnexinV+) and viable (AnnexinV−/PI−) cells. Statistical analysis was performed using one-way ANOVA. **P < 0.01 (mean ± SD, n = 3). **(G)** Representative light microscopy and fluorescence images of organoids following lentivirus infection. The green fluorescence is carried by the vector, indicating successful lentiviral infection of the organoids. Black scale bars, 100 μm. **(H)** Dose-response curves of no-target control organoids (CRC054 and CRC060), shMIEF2 and sgMIEF2 to OXL (gray connected dots: control; green connected dots: shMIEF2; blue connected dots: sgMIEF2).

To further refine our CRISPR/Cas9 knockdown library screening results and determine the potential role of MIEF2 in the OXL treatment of CRC, we constructed MIEF2 knockdown cell lines using SW480 cells. qPCR and western blotting confirmed that both shRNA and sgRNA sequences effectively silenced MIEF2 expression in SW480 cells (**Figures 4A**, **B**). We then performed CCK8 and apoptosis assays to further confirm whether the loss of MIEF2 expression leads to OXL resistance in CRC cells. After 48 h of 10 μM OXL treatment, compared to the IC_50_ values of WT cells, the IC_50_ values increased 4-fold in MIEF2 knockdown (SW480-shMIEF2) cells (mean from 0.1267 μM to 0.5518 μM) and 7-fold in MIEF2 knockout (SW480-sgMIEF2) cells (mean from 0.1267 μM to 0.9264 μM) (**Figures 4C, D**). After silencing the target gene in SW480 cells, the IC_50_ value of OXL resistance increased by 4- to 7-fold. In addition, the percentage of apoptotic cells after OXL treatment decreased from approximately 38% to 16% after knockdown or knockout of MIEF2 in SW480 compared to the no-targeting control (**Figures 4E, F**).

Subsequently, we knockdown MIEF2 expression in organoid CRC054 and CRC060 (**Figure 4G**) and then assayed cellular activity after 48 h of treatment with OXL. Compared to the control, the OXL response curves of shMIEF2 and sgMIEF groups were significantly shifted to the right, indicating that MIEF2 deletion increased the resistance of CRC054 and CRC060 to OXL (**Figure 4H**). These results suggest that the deletion of MIEF2 correlates with CRC resistance to OXL.

### Overexpression of MIEF2 partially restores CRC sensitivity to OXL

**Figure 5:**
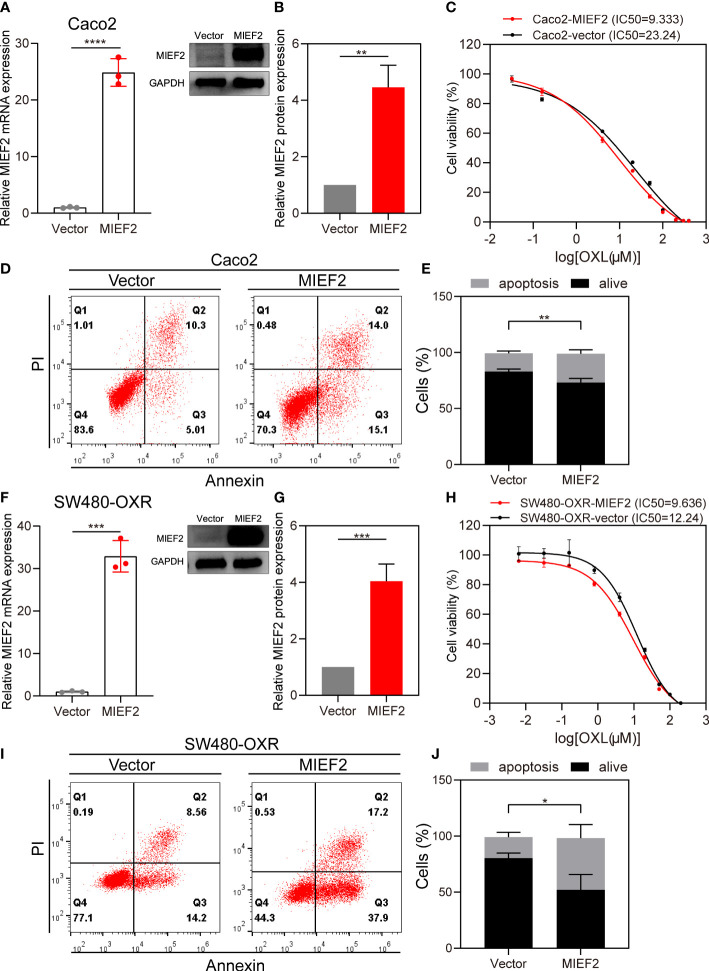
Overexpression of MIEF2 partially restores CRC sensitivity to OXL. **(A, B)** qPCR and western blotting to show overexpression of MIEF2 in Caco2. Bar plots show MIEF2 expression of vector and MIEF2 expressing clones. Statistical analysis was performed using two-tailed unpaired t-test. **P < 0.01, ****P < 0.0001 (mean ± SD, n=3 repetitions). **(C)** OXL Dose-response curves of vector and MIEF2 expressing clones in Caco2 (black connected dots: Caco2-vector, red connected dots: Caco2-MIEF2). **(D, E)** Comparison of apoptosis induction between vector- and MIEF2-transfected Caco2 cells after oxaliplatin treatment by flow cytometry analysis. Overexpression of MIEF2 upregulates cell apoptosis after OXL treatment in Caco2. Stacked plots show the percentage of apoptotic (AnnexinV+) and viable (AnnexinV−/PI−) cells. Statistical analysis was performed using two-tailed unpaired t-test. **P < 0.01 (mean ± SD, n = 3). **(F–J)** The same results as Caco2 were detected after overexpression of MIEF2 in SW480-OXR. Statistical analysis was performed using two-tailed unpaired t-test. *P < 0.05, ***P < 0.001 (mean ± SD, n = 4).

To validate the role of MIEF2 in OXL-mediated responses, we overexpressed MIEF2 in Caco2 and SW480-OXR to determine whether increasing MIEF2 expression in OXL-resistant cells could restore SW480 sensitivity to OXL treatment. We transduced the constructed plasmid expressing MIEF2 into Caco2 to generate MIEF2 stably expressed clones. The MIEF2 mRNA and protein expression were significantly increased in MIEF2 stably transduced clones compared to vector-transduced cells indicating successful generation of MIEF2 overexpression clones (**Figures 5A, B**). Vector or MIEF2-expressing Caco2 cells were treated with 10 μM OXL to detect changes in OXL drug responsiveness and apoptosis. After 48 h of OXL action, OXL IC50 values in MIEF2 overexpressing Caco2 increased approximately 2.5-fold (mean value from 9.333 μM to 23.24 μM) compared to the control (**Figure 5C**), and the average percentage of apoptotic cells increased from 16% to 26% (**Figures 5D, E**). Overexpression of MIEF2 in SW480-OXR (**Figures 5F, G**) revealed that the altered responsiveness of SW480-OXR to OXL had a similar trend to Caco2. 48 h after 10 μM OXL treatment, the CCK-8 assay revealed that the IC50 value of SW480-OXR overexpressing MIEF2 increased from 9.636 μM in the vector control to 12.24 μM (**Figure 5H**), and the percentage of apoptotic cells increased from about 19% to 46%, about a 2.5-fold increase (**Figures 5I, J**).

### MIEF2 regulates the release of mitochondrial cytochrome C in response to OXL treatment

**Figure 6:**
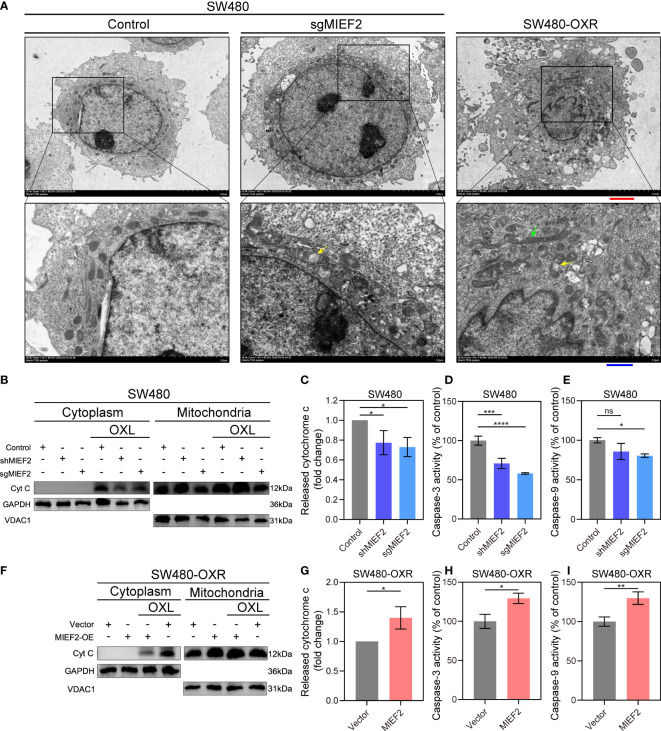
MIEF2 regulates the release of mitochondrial cytochrome c in response to OXL treatment. **(A)** Representative transmission electron microscopy images of mitochondrial in SW480 and SW480-OXR. Yellow arrowhead: mitochondrial swelling, green arrowhead: abnormal long mitochondria. Red scale bars, 2.5 μm. Blue scale bars, 1 μm. **(B)** After OXL treatment, cytoplasmic and mitochondrial fractions of SW480 were extracted and cytochrome c was detected by Western bolting. Grayscale analysis of cytochrome c content in the cytoplasm was performed to assess cytochrome c release **(C)**. In parallel, caspase- and -9 activity was assayed **(D, E)**. SW480-OXR with or without MIEF2 overexpression was treated with OXL and cytochrome c release was detected by Western bolting **(F, G)**, followed by caspase- and -9 activity using caspase-3/9 activity assay kits **(H, I)**. Bars represent mean ± SD, n = 3 repetitions. Statistical analysis was performed using two-tailed unpaired t-test. ns P < 0.05, *P < 0.05, **P < 0.01, ***P < 0.001, ****P < 0.0001.

To further investigate the changes of mitochondria after MIEF2 deletion and OXL resistance, we examined the morphological changes of mitochondria by electron microscopy (**Figure 6A**). It could be found that the mitochondria in MIEF2-deficient SW480 (SW480-sgMIEF2) had increased volume and cavitation swelling, indicating that mitochondrial division received inhibition. In addition, the increased number and apparent swelling of mitochondria in SW480-OXR, and even the appearance of aberrant long mitochondria, suggest that mitochondrial remodeling is affected during the acquisition of OXL resistance by SW480. Therefore, we hypothesized that MIEF2 deletion would inhibit mitochondrial division, contribute to mitochondrial remodeling, and inhibit mitochondrial function, thus participating in the process of SW480 acquired drug resistance.

The release of cytochrome C from the mitochondria in the cytoplasm is often used as an important indicator of apoptosis (30). To determine the regulatory role of MIEF2 in OXL-mediated cell death, we examined the release of mitochondrial cytochrome C and the activity of downstream apoptotic effector molecules, including caspase-9, and caspase-3. We observed the early apoptotic activity by detecting the release of cytochrome C. After incubation with OXL, cells exhibited reduced counts of released CYCS when cells were depleted of MIEF2 (SW480-shMIEF2, SW480-sgMIEF2) compared to control (**Figures 5B, C**). In SW480 cells with loss of MIEF2 expression, lower caspase-9 and -3 activity were observed than in the non-targeted control (**Figures 5D, E**). On the other hand, in case of SW480-OXR, the overexpression of MIEF2 prompted the release of cytochrome C (**Figures 5F,G**) and enhanced caspase-9 and -3 activity (**Figures 5H, I**). This result suggested that MIEF2 deletion inhibited apoptosis by affecting the release of mitochondrial cytochrome C, which mediated cellular resistance to OXL.

## Discussion

Previous studies have shown that the discovery of OXL resistance genes can provide ideas for novel therapeutic strategies (31). Determining the underlying mechanisms of chemoresistance in CRC is important to overcome drug resistance problems and discover new therapeutic strategies. In this study, we used the library screening strategy described by Feng Zhang (12, 26), A genome-wide knockdown screening of CRC cells treated with and without OXL led to the identification of 3736 positively enriched genes and we determined that the deletion of MIEF2 was an important driver of OXL resistance. We also demonstrated the comparability of the data by sgRNA matches and loss rates [it is perfectly normal for positive screens to have higher sgRNA loss at later time points, as some surviving clones may dominate the final pool, whereas most other cells die because of the loss of sgRNA (32)].

To further elucidate the function of MIEF2 deletion in CRC oxaliplatin resistance, we knocked down the expression of MIEF2 in SW480. The deletion of MIEF2 in SW480 enhanced the resistance of SW480 to OXL (**Figures 4A–F**). The same trend was observed in the organoids CRC054 and CRC060 (**Figures 4G, H**). We subsequently overexpressed MIEF2 in Caco2 and restored MIEF2 expression in SW480-OXR, which resulted in increased sensitivity of Caco2 and SW480-OXR to OXL (**Figure 5**). In addition, MIEF2 deletion inhibited OXL-induced apoptosis by affecting mitochondrial cristae remodeling and decreasing cytochrome c release (**Figure 6**). MIEF2 deletion may be part of a strategy for CRC cells to evade chemotherapy treatment.

Platinum generally triggers apoptosis *via* the intrinsic pathway initiated at the level of the mitochondria (7). Mitochondrial dynamics is closely related to many cellular processes including apoptosis and mitochondrial autophagy, the most prevalent mechanism being mitochondrial fission to accelerate apoptosis, whereas mitochondrial elongation is believed to protect cells from apoptosis. Several studies have shown that MIEF2 deletion inhibits apoptosis by affecting the dynamics of mitochondrial homeostasis. While the overexpression of MIEF2 isolates excess inactive DNM1L on OMM, thereby prohibiting mitochondrial fission (20, 33), the depletion of MIEF1 or MIEF2 eliminates oligomerization on the DNM1L mitochondria, leading to mitochondrial elongation or collapse (20). On the other hand, double knockdown (DKO) of MIEF1 and MIEF2 was reported to protect cells from intrinsic apoptotic progression through mitochondrial cristae remodeling (25). Notably, MIEF1 and MIEF2 may play different roles in response to apoptotic stimuli. MIEF1 has specific autoregulation of BAX-mediated cell death and is not associated with DNM1L-mediated mitochondrial fission (23). However, the MIEF2-induced mitochondrial fusion phenotype is more severe than the MIEF1-induced phenotype, wherein mutations in MIEF2 result in mitochondrial kinetic imbalances and combined respiratory chain enzyme defects in skeletal muscles (17, 34).

MIEF2 has been intensively studied in different diseases as a key molecule regulating mitochondrial division and has potential clinical value (35–38). However, the function of MIEF2 itself may be different from the combined function of MIEF1 or MIEFS, and the effect of MIEF2 on apoptosis remains largely unclear (23). Therefore, we need to know more about the relationship between mitochondrial dynamics and apoptosis, and the complex mechanism of MIEF2 involved in the process of responding to apoptosis. We identified that MIEF2 was involved in CRC responsiveness to OXL through CRISPR knockout screening and confirmed that MIEF2 loss mediated OXL resistance by inhibiting apoptosis. The loss of MIEF2 could be a potential molecular marker of OXL resistance in CRC. Additionally, the dynamic regulation of the mitochondria involved in apoptosis may provide new insights into OXL therapy. We need to perform more in-depth research in the future to determine whether MIEF2 deletion mediates OXL resistance in CRC by inhibiting mitochondrial division or independently of mitochondrial dynamics.

## Conclusions

In our study, we performed CRISPR knockdown screening to determine that MIEF2 deletion was a key driver of OXL resistance in CRC and elucidated that MIEF2 loss reduced mitochondrial cytochrome C release, thereby inhibiting endogenous apoptosis, and eventually leading to OXL resistance in CRC. In conclusion, we identified potential predictors of OXL resistance in CRC, among which MIEF2 may serve as a predictor of OXL drug responsiveness and a potential target for the development of therapies to improve chemotherapy effectiveness in CRC.

## Data availability statement

The raw sequencing data presented in this study can be found in the Sequence Read Archive (SRA) DataBase (https://www.ncbi.nlm.nih.gov/sra) with project number PRJNA864645.

## Ethics statement

The studies involving human participants were reviewed and approved by the Ethical Committee of the First Affiliated Hospital of Chongqing Medical University (2020-557). The patients/participants provided their written informed consent to participate in this study.

## Author contributions

Conception or design of the work: CX; Experimental task: CX, KL, and YL; Data collection: BT and YL; Data analysis and interpretation: CX and KH; Drafting the article: CX and XP; Critical revision of the article: AJ and WW; Final approval of the version to be published: ZW and WW. All authors contributed to writing and approved the final submitted manuscript.

## Funding

This study was supported by Chongqing key diseases Research and Application Demonstration Program (Colorectal Cancer Prevention and Treatment Technology Research and Application Demonstration [No. 2019ZX003]), Chongqing Municipal Natural Science Foundation [No. cstc2018jcyjAX0194], and the General project of Chongqing Nature Science Foundation [No. cstc2021jcyj-msxmX0283].

## Acknowledgments

We thank BMCSCI (http://www.bmcscience.com/) for editing this manuscript.

## Conflict of interest

The authors declare that the research was conducted in the absence of any commercial or financial relationships that could be construed as a potential conflict of interest.

## Publisher’s note

All claims expressed in this article are solely those of the authors and do not necessarily represent those of their affiliated organizations, or those of the publisher, the editors and the reviewers. Any product that may be evaluated in this article, or claim that may be made by its manufacturer, is not guaranteed or endorsed by the publisher.
