# Supplementary material for: CRISPR-based knockout screening identifies the loss of MIEF2 to enhance oxaliplatin resistance in colorectal cancer through inhibiting the mitochondrial apoptosis pathway
Source: Front Oncol. 2022 Aug 29;12:881487. doi: 10.3389/fonc.2022.881487 (PMC9465453; doi:10.3389/fonc.2022.881487)
Supplement: Supplementary file 1 [file DataSheet_1.zip › Supplementary File 1.docx]

**Supplemental information（tables and figures）**

**Supplemental tables**

**Table 1.** Sequences of primers for qPCR analysis and for knockdown or knockout of MIEF2 expression.

| **1. Primers used in q-PCR analysis** | | |
| --- | --- | --- |
| **Gene** | **Forward Primer** | **Reverse Primer** |
| *MIEF2* | AGCCTGCCCTGCCACTTCA | CAACCACCCGTTTCCACCC |
| *MIEF2 (CDS)* | ATGGCAGAGTTCTCCCAGAAA | CCCTGTCAATGAACCGCTT |
| *MAST3* | CCATCTTCCGGCTATGGAACC | TCAAGCACATTCTCTGAGCTG |
| *SIK2* | AGACCACCCTCACATAATCAAAC | ATTTTCGCCTGGCTTCAGACT |
| *MAPT* | CCAAGTGTGGCTCATTAGGCA | CCAATCTTCGACTGGACTCTGT |
| *HOXB13* | CCAGTTACCTGGACGTGTCTG | GGACCTGGTGGGTTCTGTTC |
| *PRKCB* | AGCCCCACGTTTTGTGACC | GCTGGGAACATTCATCACGC |
| *ACE2* | CGAAGCCGAAGACCTGTTCTA | GGGCAAGTGTGGACTGTTCC |
| *SFTPA1* | GGAGACTTCCGCTACTCAGAC | CAGTTCCTGTCATTCCACTGC |
| *PTGIR* | TTCCGCTTCTACGCCTTCAAC | ACCCAGAGCTTGAGTCGCT |
| *FASN* | AAGGACCTGTCTAGGTTTGATGC | TGGCTTCATAGGTGACTTCCA |
| *SCD1* | AGAATGGAGGAGATAAGT | TAGCAGAGACATAAGGAT |
| *HMGCS1* | GCTCTGGAATCTGGAATG | CTCTTCAATGGCAGTGTT |
| *HMGCR* | AAGAAGACAGCCTGAATAG | ATCCTCCACAAGACAATG |
| *CD36* | GGCTGTGACCGGAACTGTG | AGGTCTCCAACTGGCATTAGAA |
| *CPT1A* | ATCAATCGGACTCTGGAAACGG | TCAGGGAGTAGCGCATGGT |
| *ACOX1* | TGGCGGACATGGCTATTCTCAC | TGGCTGGGCAGGTCATTCAAG |
| *SREBP1* | ACAGTGACTTCCCTGGCCTAT | GCATGGACGGGTACATCTTCAA |
| *SREBP2* | AACGGTCATTCACCCAGGTC | GGCTGAAGAATAGGAGTTGCC |
| *GAPDH* | AGGCACCAGGGCGTGAT | GCCCACATAGGAATCCTTCTGAC |
| **2. shRNAs** | | |
| **Target** | **Forward** | **Reverse** |
| *MIEF2-sh1* | GCGCUAUACAGUGGCCUACAGGAGC | GCUCCUGUAGGCCACUGUAUAGCGC |
| *MIEF2-sh2* | AAAAACCACUAUUCAAAUCCC | GAUUUGAAUAGUGGUUUUUCU |
| **3. sgRNAs** | | |
| **Target** | **Forward** | **Reverse** |
| *MIEF2-KO1* | AGCTTGAGTTCTGCCCCCGT | ACGGGGGCAGAACTCAAGCT |
| *MIEF2-KO2* | TTGACAGGGCCACTAGCCCG | CGGGCTAGTGGCCCTGTCAA |

**Table 2.** PCR primers for generate MIEF2 DNA fragment (NM_139162).

| *MIEF2-FOR* | 5’- CGTACCAGATTACGCGGATCCATGGCAGAGTTCTCCCAGAAACG -3’ |
| --- | --- |
| *MIEF2-REV* | 5’- TGCTCGAGGCTAGCGAATTCCTAGAGCAGCCCCTCGGG -3’ |

**Table 3.** Primary antibodies used in this study.

| **Antibody** | **Company (Cat. No.)** | **Working dilutions** |
| --- | --- | --- |
| MIEF2 | Proteintech (28178-1-AP) | WB: 1/1000; IHC:1/250 |
| Cytochrome c | Proteintech (10993-1-AP) | WB: 1/2000 |
| GAPDH | Proteintech (60004-1-Ig) | WB: 1/30000 |
| VDAC1 | Proteintech (55259-1-AP) | WB: 1/1000 |

**Supplemental figures**

**
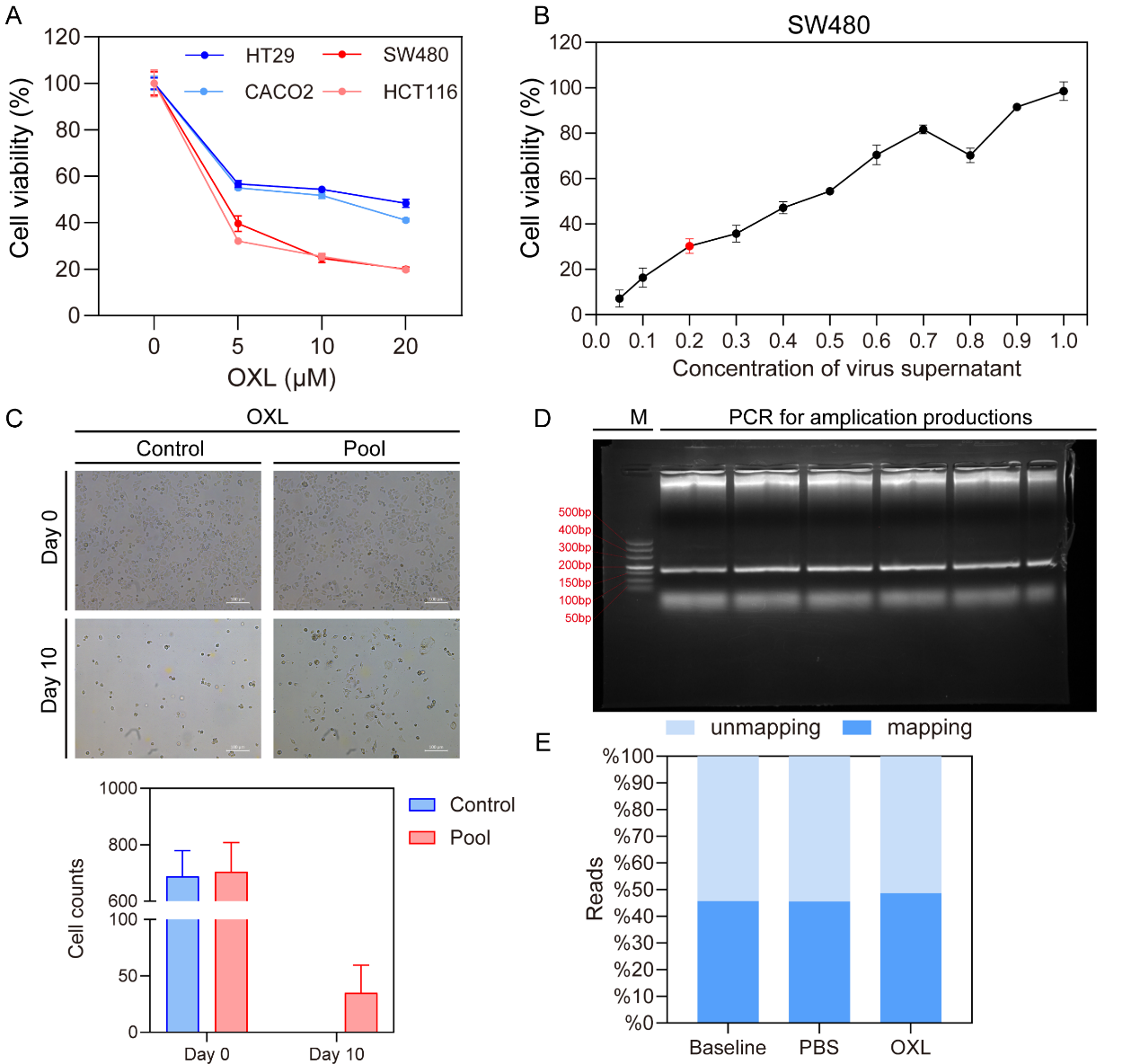
**

**Figure S1.** Mutant cell pool construction and OXL screening. **(A)** OXL dose-response curves of Caco2, HT29, HC116, SW480. Cells were incubated with different concentrations of OXL for 48h and then assayed for cell activity using CCK-8. **(B)** Determination of the lentivirus concentration required for Brunello lentiviral library to infect SW480 at 30% transfection efficiency, red dots correspond to the concentration of the final selected diluted virus. (**C**) Representative light microscopy images of 10 μM OXL treated non-targeted control and mutant cell pool on Day 0 and Day10, with control on the left and mutant cell pool on the right. Bar graph shows the average cell counts of the 3 fields of view between the different groups. (**D**) PCR amplification of the sgRNA region used for deep sequencing analysis, as indicated by electrophoresis. M: 500 bp DNA marker. Forward and backward primers used were NGS-F (5’-TTGTGGAAAGGACGAAACACCG-3’) and NGS-R, respectively.，NGS-R (5’-CCAATTCCCACTCCTTTCAAGACCT-3’). Baseline group, PBS group, and OXL group sequencing results of sgRNA matching **(E)**.

**
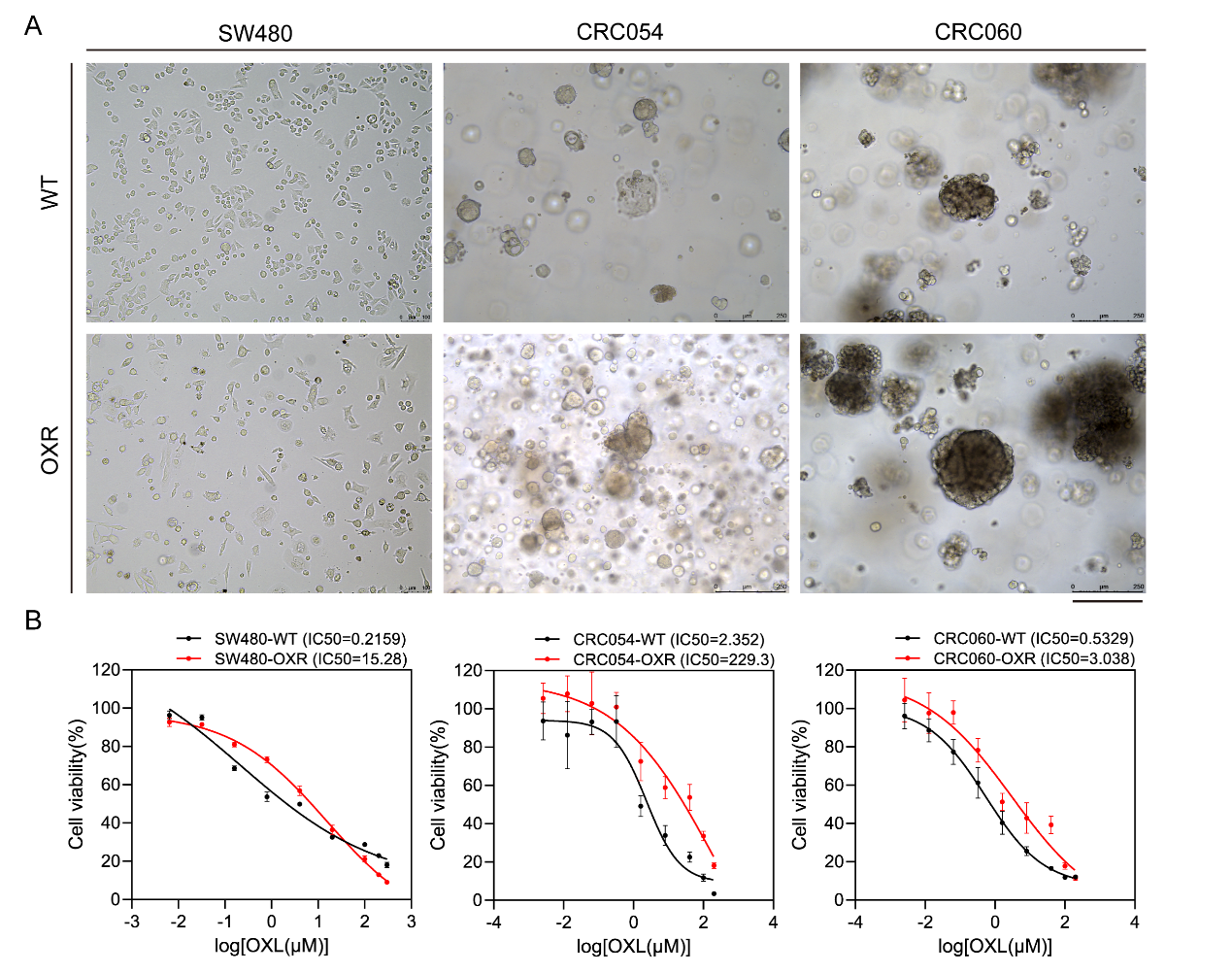
**

**Figure S2. Construction of OXL resistance models. (A)** Representative light microscopic images of SW480-OXR, CRC054-OXR, CRC060-OXR and their parental cells or organoids. Scale bars: 250 μM. **(B)** Dose-response curves of SW480-OXR, CRC054-OXR, CRC060-OXR and their parental cells or organoids were assayed for cell viability after culturing with medium containing different concentrations of OXL for 48 h. The IC50 value of OXL in SW480-OXR was approximately 70-fold higher than WT cells, approximately 97-fold higher in CRC054-OXR, and approximately 5-fold higher in CRC060-OXR, indicating that SW480-OXR, CRC054-OXR, and CRC060-OXR acquired resistance to OXL.
